# Obstetric conditions requiring intensive care at the Analankininina University Hospital Centre, Toamasina, Madagascar

**DOI:** 10.11604/pamj.2026.53.179.52073

**Published:** 2026-04-30

**Authors:** Rafanomezantsoa Toky Andriamahefa, Razafindrabekoto Lova Dany Ella, Razafindraibe Felanarivo Andriatompoina, Razermera Andy Harson, Harioly Nirina Marie Osé Judicaël, Andriatahina Todisoa Nomenjanahary

**Affiliations:** 1 Faculty of Medicine, University of Toamasina, Toamasina, Madagascar,; 2 Multidisciplinary Intensive Care Unit, Analankininina University Hospital Centre, Toamasina, Madagascar,; 3 Faculty of Medicine, University of Fianarantsoa, Fianarantsoa, Madagascar,; 4 Department of Obstetrics and Gynaecology, Analankininina University Hospital Centre, Toamasina, Madagascar,; 5 Faculty of Medicine, University of Antsiranana, Antsiranana, Madagascar

**Keywords:** Intensive care, obstetric complications, maternal morbidity, Madagascar

## Abstract

The management of obstetric emergencies in the intensive care unit (ICU) requires a multidisciplinary approach. This study aimed to describe the epidemiological and clinical profiles of obstetric patients admitted to the ICU of Analankininina University Hospital Centre (CHUAT). This was a retrospective descriptive study conducted over an 18-month period, from January 1, 2023, to June 30, 2024. A total of 58 obstetric cases were recorded, representing a prevalence of 10.8%. The mean age was 25.29 ± 6.73 years. Hypertension was the most common medical history. Direct admissions accounted for 86.8% of the patients. Postoperative cases constituted the main reason for admission (48%), followed by complications of pregnancy-induced hypertension (26%). Four maternal deaths were reported, mainly due to disseminated intravascular coagulation, septic shock, and hypovolemic shock. The management of critically ill obstetric patients plays a key role in reducing maternal mortality. Establishing a dedicated obstetric and gynecologic ICU would improve access to care for near-miss cases and enhance outcomes.

## Introduction

The obstetric intensive care unit provides specialised management for women requiring close monitoring or critical care interventions. At the Analankininina University Hospital Centre, Toamasina (CHUAT), the multidisciplinary intensive care unit also manages major obstetric emergencies such as eclampsia and postpartum haemorrhage [1]. In high-income countries, obstetric patients represent about 1% of intensive care admissions, most commonly for cardiac disease, severe haemorrhage, infection, or shock [2]. Approximately 0.5% of pregnant women require intensive care, and 30-50% need mechanical ventilation [3]. However, maternal morbidity and mortality remain disproportionately high in low-resource settings, reflecting socioeconomic and organisational disparities rather than medical incompetence [4-6]. In Madagascar, limited data are available on obstetric admissions to intensive care. At CHUAT, no detailed statistics had previously been reported [7,8]. This study therefore aimed to describe the epidemiological and clinical profiles, as well as outcomes, of obstetric patients admitted to the multidisciplinary intensive care unit during 2023-2024.

## Methods

We conducted a retrospective, descriptive, cross-sectional study including women admitted to the multidisciplinary intensive care unit of the Analankininina University Hospital Centre (CHUAT) over an 18-month period, from January 2023 to June 2024. The study population consisted of all female patients admitted to the intensive care unit during the study period. All complete medical records of patients aged over 15 years were included, while incomplete files were excluded. The variables analysed included epidemiological characteristics, obstetric history, reasons for emergency admission, type of surgical intervention, indication for intensive care admission, length of hospital stay, and patient outcomes. Data were collected from medical records. Data entry was performed using Microsoft Word 2021®, and tables were created with Microsoft Excel 2021® and Epi-Info version 3.5.3. Ethical principles, including confidentiality, anonymity, and professional secrecy, were strictly observed. No patient names were recorded, and a coding system was used to ensure data protection. The retrospective and single-centre design represents the main limitation of this study.

## Results

During the study period, 537 patients were admitted to the multidisciplinary intensive care unit, including 58 cases (10.8%) of obstetric pathologies. The mean age of the women admitted was 25.3 ± 6.7 years, with a predominance of young women aged 15-25 years (42.6%). Most of the admitted women (81.5%) were employed in the primary sector. The mean gravidity was 2.7. Primigravidae accounted for 51.7%, multigravidae for 46.4%, and nulligravidae for 1.9%. The mean parity was 1.8, with primiparous women representing 42.6% and nulliparous women 26.8% of admissions. Hypertension was the most common medical history (18.5%), including 6.4% with a prior history during pregnancy. Regarding the mode of admission, 86.8% (50/58) of patients presented directly to the emergency department, 12.8% were referred from other facilities, and 0.4% were interdepartmental transfers. At admission, 17.7% of patients had a Glasgow Coma Scale (GCS) alteration, including nine cases in coma (Table 1). The categories of ICU admissions were diverse (Table 2). Most patients were postoperative cases (48%), followed by postpartum complications (19%) requiring transfer to intensive care. Among surgical indications, eclampsia was the most common reason for caesarean section (n = 12; 60%). Pelviperitonitis was the leading indication for laparotomy (n = 3; 37.5%), followed by hysterectomy, ruptured ectopic pregnancy, and infectious adhesions. Postpartum complications requiring intensive care were mainly postpartum haemorrhage (n = 3) and severe infections (n = 3), including sepsis (n = 2) and post-abortion meningitis (following induced abortion). Medical conditions accounted for some ICU admissions, primarily asthma exacerbations (25%) and vaso-occlusive crises (75%) among pregnant women with sickle-cell disease. Among hypertensive disorders of pregnancy, eclampsia (40%) was the leading cause of ICU admission, followed by placental abruption (13.3%), acute pulmonary oedema (26.7%), and hemolysis, elevated liver enzymes and low platelets (HELLP) syndrome (20%). The mean length of stay in intensive care was 4 ± 3.5 days (range: 1-10 days). Regarding outcomes, 81.0% of patients had a favourable recovery, 6.8% died, 8.6% developed complications, and 3.4% required transfer to another speciality. Among the four deaths, two were due to hypovolaemic shock secondary to postpartum haemorrhage, one to disseminated intravascular coagulation (DIC), and one to septic shock.

**Table 1:** distribution of women according to the Glasgow score at admission

Glasgow coma scale	Effectif (n=58)	Pourcentage (%)
G (3-7)	9	15.47%
G (7-13)	1	2.30%
G >13	48	82.30%
**Total**	58	100%

**Table 2:** distribution according to the reasons for admission to the intensive care unit

Reasons for admission	Number (n=58)	Percentage (%)
Postoperative cases	28	48%
Postpartum complications	11	19%
Medical conditions	4	7%
Hypertensive complications	15	26%
**Total**	58	100%

## Discussion

The mean age of patients admitted was 28.4 years, with a predominance of young women aged 15-25 years (42.6%). This result is comparable to those reported by Bonnet *et al*. in France (25 years) [2] and Owono *et al*. in Cameroon (25.7 ± 7.3 years) [9]. The most represented age group in previous studies was 20-39 years [2]. Similarly, Daddy *et al*. in 2014 in Niger found a predominance of women aged 30-36 years [10]. According to Owono *et al*. study in Cameroon, the mean age was 25.7 ± 7.3 years [9]. Our results differ from those of Zeeman *et al*. in Bamako (2009), where the 14-19-year age group predominated [11]. The majority of women (81.5%) were employed in the primary sector, of whom 67.2% were housewives. These findings partially align with those of Owono *et al*. [9], but differ from Tchaou *et al*. in Benin [12] and Zeeman *et al*. in Bamako [11], who reported a more diversified occupational distribution. The low socioeconomic and educational level remains a major risk factor for obstetric complications.

Primigravidae (51.7%) and primiparous women (42.6%) were predominant, rates similar to those observed by Tchaou *et al*. [12] and Owono *et al*. [9], but higher than those reported by Andrianirina *et al*. in Antananarivo [8]. Regarding medical history, 82.6% of patients had no history of abortion, and 71.3% had no prior medical condition. Hypertension was the most common antecedent (18.5%), similar to findings by Tchaou *et al*. [12] and Andrianirina *et al*. [8]. In our study, 82.6% of women had never had an abortion. Harioly *et al*. at Befelatanana Maternity, Antananarivo, found that 17.2% of women with a previous abortion experienced pregnancy complications [13]. The main reasons for admission were genital bleeding (28.3%), abdominal pain (20.8%), and seizures (14.7%). In the intensive care unit, eclampsia was the leading cause (15.5%), followed by operated ectopic pregnancies (12.8%) and complicated post-caesarean cases (9.4%). These results differ from those of Owono *et al*. [9] and Tchaou *et al*. [12], who reported predominance of hypertensive and haemorrhagic emergencies. At admission, 17.8% of patients presented with an altered Glasgow Coma Scale, including 15.5% in coma-lower than the 23% reported by Samaké *et al*. at the Gabriel Touré University Hospital [14]. The mean length of stay was 4 days, comparable to results from Tchaou *et al*. [12] and Mangané *et al*. [15]. Complications occurred in 12.1% of cases, mainly haemorrhagic (6.7%), and were most frequent among women under 35 years, multiparous, or multigravidae, consistent with Mangané findings [15]. The overall mortality rate was 6.8%, lower than that reported in other African series. Hypovolaemic shock and eclampsia-related complications each accounted for 33.3% of deaths, followed by septic shock (11%). These results are consistent with those of Owono *et al*. [9], highlighting the persistence of avoidable causes of maternal death. These findings are consistent with previous studies on obstetric critical care, highlighting the need for specialised management and early intervention to improve maternal outcomes [11]. Economic hardship, delayed referral, inadequate technical facilities, and poor antenatal follow-up remain major determinants of maternal morbidity and mortality in low-resource settings.

## Conclusion

Admission of a patient to intensive care for pregnancy-related complications requires coordination, multidisciplinary collaboration, and clinical experience. Maternal pathologies managed in obstetric and general intensive care units are highly diverse, and the management of critically ill obstetric patients demands both technical competence and organisational efficiency. Developing structured protocols for quality and safety of care within maternity services, supported by multidisciplinary high-risk medical teams, is essential for achieving hospital accreditation and improving outcomes. The maternal condition at admission and the associated complications remains the main determinants of maternal outcome.

### What is known about this topic

- Severe obstetric complications remain a major cause of admission to intensive care units in low-resource settings;
- Hypertensive disorders of pregnancy and postpartum haemorrhage are among the leading causes of maternal morbidity and mortality in Africa;
- Access to intensive care and early referral are essential factors influencing maternal outcomes.

### What this study adds

- This study provides the first data on obstetric admissions to the intensive care unit at Analankininina University Hospital Centre in Toamasina, Madagascar;
- Postoperative complications and hypertensive disorders were the main indications for intensive care admission in our setting;
- Most maternal deaths were related to preventable causes, highlighting the need to improve referral systems and critical care management.

## References

[1] Lelong E, Pourrat O, Pinsard M, Goudet V, Badin J, Mimoz O (2013). Admission of women to an intensive care unit during pregnancy or the postpartum period: circumstances and prognosis. A retrospective series of 96 cases. Rev Med Interne.

[2] Bonnet MP, Chantry A, Seco A, Chiesa-Dubruille C, Fresson J, Bouvier-Colle MH (2015). Admissions obstétricales en réanimation: des leçons à tirer pour l´organisation des soins. Anesthésie & Réanimation.

[3] >Benhamou D Evolution of safety in obstetric anaesthesia in France.

[4] Say L, Chou D, Gemmill A, Tunçalp Ö, Moller AB, Daniels J (2014). Global causes of maternal death: a WHO systematic analysis. Lancet Glob Health.

[5] Fourrier F (2007). Pathologie obstétricale en réanimation. Des généralités aux principes. Réanim.

[6] Munur U, Karnar D, Gunpun T (2005). Critically ill obstetric patients in an American and an Indian public hospital: comparison of case-mix, organ dysfunction, intensive care requirements, and outcomes. Intensive care medicine. Intensive Care Med.

[7] Abdoul AD, Moussa D, Magatte M, Sokhna DS, Marie EF, Jean CM (2013). Epidemiological profile and management of eclampsia in Senegal: about 62 cases. Pan Afr Med J.

[8] Andrianirina M, Harioly NMOJ, Rasolonjatovo TY, Rabearivony N, Andrianampanalinarivo HR, Randriamiarana JM (2009). Profil épidémiologique des cardiaques gestantes passées en réanimation. Revue d´Anesthésie-Réanimation et de Médecine d´Urgence.

[9] Owono EP, Metogo MAJ, Tchokam L, Danwang C, Kago TL, Afane EA (2017). Complications obstétricales admises en réanimation: épidémiologie, diagnostic et pronostic. Health Sciences and Diseases.

[10] Daddy H, Adehossi E, Gagara M, Bako Maiga AF, Foumakoye GA, Sani R (2014). Profil épidémiologique des patients admis au service de réanimation de l´Hôpital National de Niamey-Niger. SARAF.

[11] Zeeman GG (2006). Obstetric critical care: a blueprint for improved outcomes. Crit Care Med.

[12] Tchaou BA, Salifou NFMHK, Chobli EZM (2015). Obstetric emergencies at the University Hospital of Parakou, Benin: clinical, therapeutic, and outcome aspects. Eur Sci J.

[13] Harioly NMO, Rasolonjatovo TY, Andrianirina M, Randriambololona DMA, Ranoaritiana DB, Andrianjatovo JJ Epidemiological profile of preeclamptic and eclamptic women admitted to intensive care.

[14] Samaké BM, Togo A, Diallo B, Almenoun A, Touré MK, Togola M (2016). Patient of resuscitation to the Center hospitable academic Gabriel Touré: clinical and prognostic aspects. Rev Afr Anesth Mé d Urg.

[15] Mangané M, Almeimoune A, Diop ThM (2025). Obstetric Complications in the General ICU at CHU Gabriel Touré, Bamako Management and Prognostic Factors. EAS J Anesthesiol Crit Care.

